# First proton minibeam radiation therapy treatment plan evaluation

**DOI:** 10.1038/s41598-020-63975-9

**Published:** 2020-04-27

**Authors:** P. Lansonneur, H. Mammar, C. Nauraye, A. Patriarca, E. Hierso, R. Dendale, Y. Prezado, L. De Marzi

**Affiliations:** 1 Institut Curie, PSL Research University, Radiation Oncology Department, Proton Therapy Centre, Centre Universitaire, 91898 Orsay, France; 2 Institut Curie, PSL Research University, University Paris Saclay, Inserm U 1021-CNRS UMR 3347, 91898 Orsay, France

**Keywords:** Radiotherapy, Biological physics

## Abstract

Proton minibeam radiation therapy (pMBRT) is a novel dose delivery method based on spatial dose fractionation. pMBRT has been shown to be promising in terms of reduced side effects and superior tumour control in high-grade glioma-bearing rats compared to standard irradiation. These findings, together with the recent optimized implementation of pMBRT in a clinical pencil beam scanning system, have triggered reflection on the possible application to patient treatments. In this context, the present study was designed to conduct a first theoretical investigation of the clinical potential of this technique. For this purpose, a dedicated dose engine was developed and used to evaluate two clinically relevant patient treatment plans (high-grade glioma and meningioma). Treatment plans were compared with standard proton therapy plans assessed by means of a commercial treatment planning system (ECLIPSE-Varian Medical systems) and Monte Carlo simulations. A multislit brass collimator consisting of 0.4 mm wide slits separated by a centre-to-centre distance of 4 or 6 mm was placed between the nozzle and the patient to shape the planar minibeams. For each plan, spread-out Bragg peaks and homogeneous dose distributions (±7% dose variations) can be obtained in target volumes. The Peak-to-Valley Dose Ratios (PVDR) were evaluated between 9.2 and 12.8 at a depth of 20 mm for meningioma and glioma, respectively. Dose volume histograms (DVHs) for target volumes and organs at risk were quantitatively compared, resulting in a slightly better target homogeneity with standard PT than with pMBRT plans, but similar DVHs for deep-seated organs-at-risk and lower average dose for shallow organs. The proposed delivery method evaluated in this work opens the way to an effective treatment for radioresistant tumours and will support the design of future clinical research.

## Introduction

The treatment of radioresistant tumours, such as high-grade gliomas or osteosarcomas, tumours situated close to a vital structure, and paediatric cancers, remains limited by the side effects of radiation such as necrosis and cognitive impairment. Proton minibeam radiation therapy (pMBRT) is a recently proposed^1,2^ innovative radiotherapy approach, which has already demonstrated a significant reduction in normal tissue toxicity in both skin^2,3^ and brain^4^, compared to standard broad-beam radiation therapy. Equivalent or superior tumour control to that obtained by standard proton therapy has been observed in glioma-bearing rats^5,6^. These results, together with the recent implementation of pMBRT in the Orsay proton therapy centre (ICPO) pencil beam scanning system^7^, have triggered reflection on the possible evaluation of this technique in phase I/II clinical trials. In this context, we have developed a first Monte Carlo-based dose calculation engine to evaluate possible treatment plans. As the main targets of pMBRT will initially be neurological, *i.e*. tumours and lesions that can be stabilised against pulmonary and/or cardiac cycles, the present study focussed on possible treatment plans for two different brain tumours, glioma and meningioma, in virtual patients derived from anonymized human patient imaging records. pMBRT treatment plans were compared to standard (seamless) proton therapy plans. To the best of our knowledge, this study represents the first complete dosimetric evaluation in human patients for such small proton field sizes using a clinical setup.

## Materials and Methods

### Dose calculation

The Monte Carlo (MC) simulation toolkit, TOPAS v3.2.0, based on Geant4.10.05p01^8^, was parameterized to model the pMBRT setup at the ICPO gantry beamline, as described in^7^. A 65 mm thick multislit brass collimator was placed between the nozzle and the patient to shape the planar minibeams. The width of the slits used to shape the minibeams was 400 µm at the collimator, which corresponds to a FWHM of around 2 mm after the first few cm of tissue. The slits were separated by a centre-to-centre distance (ctc) of 4 mm. TOPAS simulations were run with a physics list composed of seven modules: “g4em-standard_opt3”, “g4h-phy_QGSP_BIC_HP”, “g4decay”, “g4ion-binarycascade”, “g4h-elastic_HP”, “g4stopping” and “g4radioactivedecay”. This physics list is derived from that described in reference^9^ with some module name changes in line with name changes in the Geant4 code. Uncertainty was defined as the average statistical uncertainty in voxels with a dose greater than 90% of the maximum dose and was estimated from the square root of the average variance of the voxels. A total of about 10^10^ proton histories were simulated. Global uncertainty was 3% for MC-generated distributions. The range cut for all particles was set at 0.05 mm and dose scoring grid resolution was 0.5 × 0.5 × 0.5 mm^3^. All other parameters used default options. Pencil beam spot weights (expressed in monitor units [MU] per spot) and positions were calculated by the ECLIPSE treatment planning system (TPS) (Varian Medical Systems) using the Nonlinear Universal Proton Optimizer algorithm (NUPO). We applied the methodology described in^10^ to translate TPS information into number of protons per spot for the MC simulation.

Table 1 summarizes the treatment plans evaluated in this study. A pencil beam algorithm (Varian ECLIPSE software version 15.6.03) and TOPAS Monte Carlo simulations were both considered for standard (seamless) irradiation. The dose scoring grid resolution for the pencil beam algorithm was 1 ×1 ×1 mm^3^, currently used for Pencil Beam scanning (PBS) proton therapy at ICPO. A homogeneous dose to the planning target volume (PTV) region was prescribed for each field during the spot optimization process. This plan was then recalculated with the Monte Carlo simulation to allow fair comparison with pMBRT plans. The dose distributions calculated with ECLIPSE and generated by MC for conventional PBS proton therapy were compared using a 3%/3 mm local gamma index analysis.

**Table 1:** Treatment plans evaluated in this study. For each plan, the dose was delivered using a pencil beam scanning (PBS) system. Proton minibeams (pMBRT) were shaped with a multislit collimator.

	Plan	Technique	Calculation method	ctc distance
Glioma	1	Standard PT (PBS)	ECLIPSE	—
	2	Standard PT (PBS)	MC	—
	3	pMBRT	MC	4 mm
	4	pMBRT	MC	6 mm
Meningioma	1	Standard PT (PBS)	ECLIPSE	—
	2	Standard PT (PBS)	MC	—
	3	pMBRT	MC	4 mm
	4	pMBRT	MC	6 mm

Although tumour control can be achieved with highly heterogeneous dose distributions (PVDR around 6.5)^5^, one of the constraints defined for this first evaluation was to obtain a uniform distribution in the planning target volume (PTV) region, as the best results in terms of tumour control in small animal experiments were achieved with this configuration^6^. pMBRT plans used the same number of arrays (fields) as standard proton therapy (PT) plans.

Two configurations were considered in our study: a narrow centre-to-centre (ctc) distance of 4 mm, providing a uniform dose distribution in the PTV, as measurements in water showed that it is possible to create a SOBP at a depth of ~90 mm, which roughly corresponds to the average field-specific PTV depth (including range shifter thickness) investigated in our two cases, and a larger ctc distance of 6 mm to increase spatial fractionation in normal tissues (at the cost of PTV homogeneity).

Although target dose uniformity is usually a default objective during inverse planning, biologically based models in clinical treatment planning now allow users to generate highly non-uniform dose distributions depending on spatially varied biological information (supported by tumour heterogeneity, stereotactic or partial tumour boosts experience)^11^. Better tumour control rates have also been obtained with pMBRT and a PVDR of 1.2 in the target compared to standard PT^6^.

Dose-volume histograms (DVH) were calculated and compared for each plan.

### Clinical cases

Two relevant cases were selected from the ICPO clinical patient database: a high-grade glioma (in the right parietal lobe) and a meningioma (in the sphenoid wing). The patient computed tomography (CT) datasets with delineated structures used in this study were selected based on:

i. The depth of planning target volumes (PTVs). Deep-seated volumes were selected in order to assess whether spatial fractionation of the dose could be maintained in normal tissues with PVDR comparable to preclinical settings, while ensuring homogenisation at the target;
ii. The large sizes of PTV for the same reason as in (i) and to verify whether homogenisation can be maintained without needing to change the collimator in very large targets;
iii. The proximity of OAR (e.g. brainstem and right lobe) in both cases.

Treatment was delivered with 3 fields for the glioma and 2 fields for the meningioma. Patient data and treatment specifications for each field are summarised in Table 2. All procedures involving patient data (fully anonymized) were in accordance with the ethical standards, guidelines and regulations of the Institut Curie ethics committee and with the 1964 Helsinki declaration and its later amendments or comparable ethical standards (approval number DATA190299^12^). Since gathered patient data was retrospective and did not directly involve the human participants during this theoretical work, informed consent is not applicable to this study.

**Table 2:** Field specifications for the two cases investigated.

	Glioma			Meningioma	
CT dimensions (mm)	383 × 320 × 320			357 × 320 × 320	
PTV volume (cm^3^)	66.8			13.5	
Beam name	Field 1	Field 2	Field 3	Field 1	Field 2
Number of spots	1368	630	1442	515	218
Energy range (MeV)	100–150	110–160	110–150	115–150	115–150
Gantry angle (deg)	310	40	270	255	90
Patient support angle (deg)	0	0	90	90	0
Collimator-isocentre distance (cm)	14.1	14.3	13.6	14	15.2
Range shifter thickness (mm)	30	0	30	0	65
PTV minimum- maximum depth (mm)	55–100	95–165	70–100	95–135	50–80

### Dosimetric properties and metrics

**Figure 1:**
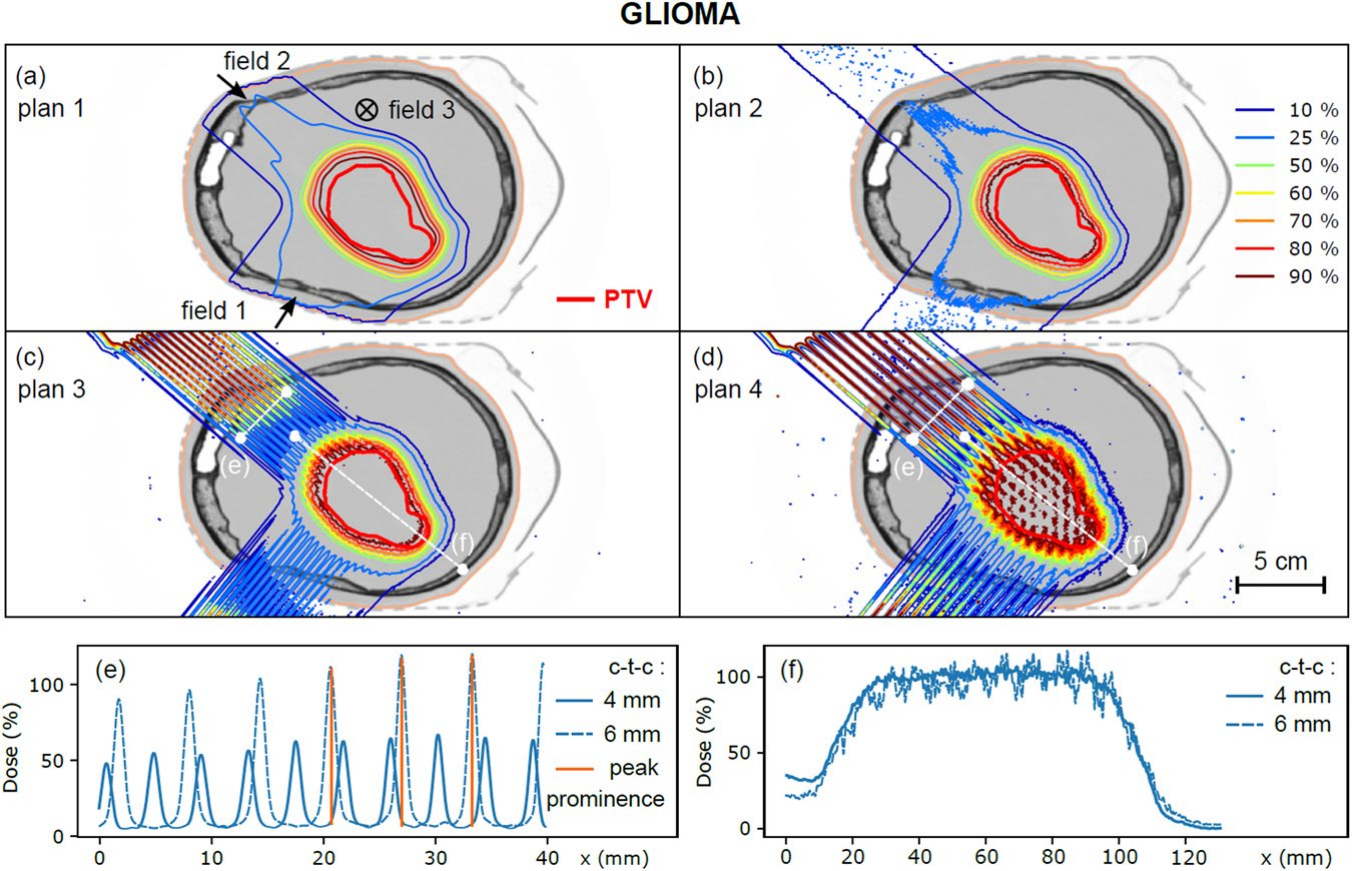
(**a**–**d**) Dose distributions calculated for plans 1 to 4 (glioma). Dose profiles are plotted in the healthy tissue region (**e**, depth 3 cm) and in the target volume (**f**) for plans 3 and 4. The prominence of the peaks is displayed in orange for the ctc of 6 mm.

Contrary to conventional radiotherapy, minibeam radiation therapy dose profiles are not flat, but composed of high-dose areas, called peaks, and low-dose areas, called valleys. The ratio between these two doses, called the peak-to-valley dose ratio (PVDR), is an important dosimetric parameter in the context of pMBRT, as high PVDR values and low valley doses are required to ensure tumour control and healthy tissue sparing, respectively^13,14^. However, evaluating and reporting the PVDR in a patient may be challenging due to the marked inhomogeneity of the 3D dose distribution. We therefore introduced the concept of dose prominence, defined as the dose difference between a peak and its lowest contour line, as illustrated in Fig. 1(e). This quantity, extensively used in topography, measures how much a peak stands out from the surrounding signal baseline. PVDR was calculated for each peak individually. For a collection of peaks, we also defined uncertainty as the standard deviation of these PVDR values. The PVDR was then calculated for each organ delineated as the ratio between the peak dose and the peak dose subtracted from its prominence. Dose homogeneity in the PTV region was evaluated using the sigma-index (s*-*index), defined as the standard deviation of the normalized differential DVH curve^15^.

### LET and RBE distribution

As the linear energy transfer (LET) distributions may be useful for interpretation of radiobiological experiments, the dose-averaged LET was computed using the method described by reference^16^ and implemented in TOPAS. The LET, computed for each step by dividing the energy deposited by the step length, was then multiplied by the energy deposited during that step. After simulation, the sum of these energy deposited-weighted LET values was finally divided by the total energy deposited for each voxel to obtain the dose-averaged LET. Increased LET values can be expected in the lateral penumbra regions of small proton beams that could result in higher RBE (relative biological efficiency)-weighted doses, an effect that can be accentuated in valley regions when a shaping device and mechanical collimation are used. In the absence of sufficient experimental data to establish an RBE model for proton minibeam radiotherapy, a first approximation of the RBE-weighted dose distributions was calculated using two different linear quadratic models. In the model proposed by Wedenberg et al^17^., RBE evolution is driven by the equation:

$$RB{E}_{Wedenberg}=\frac{1}{2D}\left(\,\sqrt{{\left(\frac{\alpha }{\beta }\right)}^{2}+4D\left(\frac{\alpha }{\beta }+0.434\,LET\right)+4{D}^{2}}-\frac{\alpha }{\beta }\right).$$

In the second model, proposed by McNamara *et al*.^18^, it is assumed that the RBE at high dose depends on the LET, such that the RBE equation is written as:

$$RB{E}_{McNamara}=\frac{1}{2D}\left(\,\sqrt{{\left(\frac{\alpha }{\beta }\right)}^{2}+4D\left(\frac{\alpha }{\beta }\right)\left(0.999064\,+\frac{0.35605}{\alpha /\beta }LET\right)+4{D}^{2}{(1.1012+0.0038703\sqrt{(\alpha /\beta )}LET)}^{2}}-\frac{\alpha }{\beta }\right)$$

where D and LET are the dose and LET matrices, respectively. As in reference^17^, α/β ratios of 10 Gy and 3 Gy were used for target volume and normal tissues, respectively.

## Results

**Figure 2:**
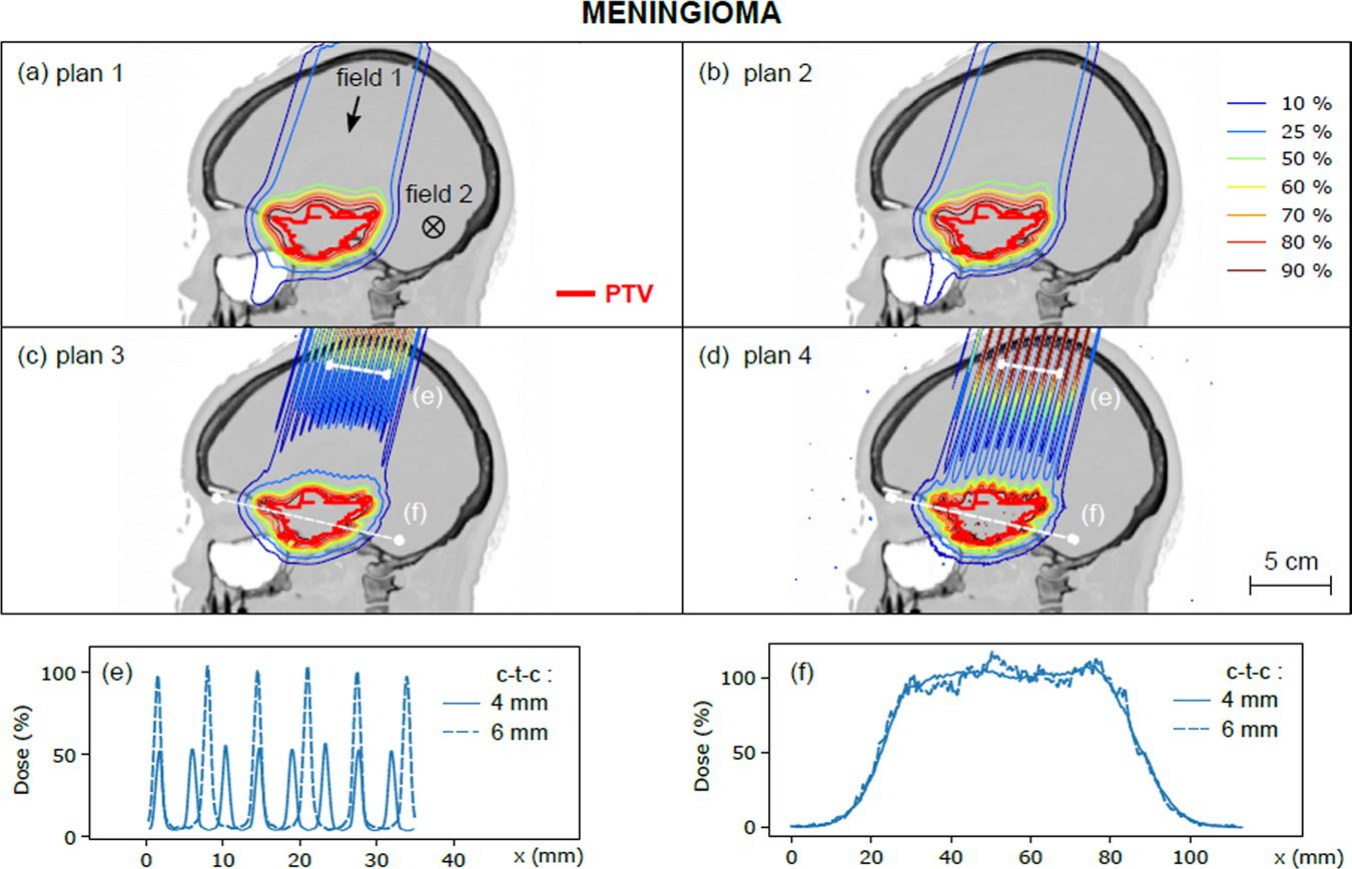
(**a**–**d**) Dose distributions calculated for plans 1 to 4 (meningioma). Dose profiles are plotted in the healthy tissue region (**e**, depth 3 cm) and in the target volume (**f**) for plans 3 and 4.

Figures 1 and 2 show the treatment plans (plans 1 to 4, see Table 1) for the glioma and the meningioma, respectively. Gamma index passing rate for the comparison of conventional plans with ECLIPSE and MC calculations, defined as the fraction of voxels with a gamma index less than 1, was 96.6% for the glioma and 98.5% for the meningioma. Dose profiles in normal tissues (at a depth of 3 cm) and in the PTV are also depicted. For a ctc of 4 mm, the PVDR of dose profiles in normal tissues was 8.0 ± 1.4 (glioma) and 8.0 ± 0.8 (meningioma), with valley doses representing 5% of the prescribed dose to the PTV. Good coverage of the PTV by the 90% isodose was observed for treatment plans 1 to 3, while heterogeneities were observed for treatment plan 4 (ctc distance: 6 mm). For a ctc of 4 mm, the PVDR evaluated in the PTV region was 1.04 ± 0.12 for the glioma and 1.05 ± 0.12 for the meningioma.

PVDR values and homogeneity indices (sigma index) evaluated in the PTV for each distribution are presented in Table 3a. PVDR values were close to 1 for all plans, while the sigma index ranged from 2.2% for plan 1 to 11.2% for plan 4 (glioma) and from 3.7% for plan 1 to 9.4% for plan 4 (meningioma), which is expected, as this index depends on DVH spread^15^. A reasonable homogeneity was achieved for plan 3 in both cases (s-index of 5.5% and 7.0% for glioma and meningioma, respectively).

**Table 3:** (a) Dosimetric homogeneity indices in the PTV calculated for treatment plans 1 to 4 (all fields). The volume (%) that receives more than 110% and less than 93% of the prescribed dose (D_50_), V_93_ and V_110_ respectively, are also given. (b) PVDR and s-index in the PTV evaluated for treatment plans 3 and 4 (individual fields). The PVDR uncertainties are the standard deviation of the PVDR, as defined in section II.3.

Plan No.	Glioma					Meningioma				
	PVDR (PTV)	s-index (%)	D_95_ (%)	V_93_ (%)	V_110_ (%)	PVDR (PTV)	s-index (%)	D_95_ (%)	V_93_ (%)	V_110_ (%)
(a)										
1	1.05 ± 0.06	2.2	98	0	0	1.06 ± 0.08	3.7	94	1.0	0
2	1.03 ± 0.04	3.3	97	0	0	1.04 ± 0.08	5.4	91	7.6	0.3
3	1.04 ± 0.08	5.5	93	2.0	0.2	1.05 ± 0.12	7.0	91	12.2	3.4
4	1.13 ± 0.16	11.2	88	14.5	7.8	1.05 ± 0.14	9.4	88	20.2	15.7
	**Plan 3**				**Plan 4**					
	**Glioma**		**Meningioma**		**Glioma**		**Meningioma**			
	**PVDR (PTV)**	**s-index (%)**	**PVDR (PTV)**	**s-index (%)**	**PVDR (PTV)**	**s-index (%)**	**PVDR (PTV)**	**s-index (%)**		
**(b)**										
Field 1	1.06 ± 0.12	8.2	1.04 ± 0.12	8.7	1.2 ± 0.3	16.5	1.09 ± 0.23	15.3		
Field 2	1.10 ± 0.14	9.8	1.06 ± 0.14	8.2	1.3 ± 0.4	23.3	1.06 ± 0.16	11.0		
Field 3	1.06 ± 0.10	6.7	—	—	1.2 ± 0.2	15.7	—	—		

The PVDR and s-index evaluated in the PTV for individual fields for treatment plans 3 and 4 are detailed in Table 3b.

In plan 3, similar levels of homogeneity in the PTV were obtained with one of the fields with the narrow ctc distance (field 3 for glioma and field 2 for meningioma) as with the complete plan. The sigma-index in plan 4 ranged between 15.7% and 23.3% for individual fields, but decreased to 11.2% for the sum of all fields (glioma). The individual field s-indices for the meningioma were 11% and 15.3% and decreased to 9.4% for the summed distribution. This result illustrates how homogeneity can be enhanced by the use of multiple irradiation fields. The PVDR was close to 1 (compatible with error bars) for each field in plans 3 and 4.

PVDR values and valley doses evaluated at different depths for treatment plans 3 and 4 are shown in Table 4. PVDR values were evaluated on fields with similar parameters (range shifter thickness, number of spots, range of energies): field 1 for the meningioma and field 2 for the glioma. These values are consistent with a previous set of measurements performed in a water phantom with a ctc distance of 4 mm and a slit width of 400 µm^7^, ranging from 9.2 to 12.8 at 20 mm and around 1.3 at 80 mm. The PVDR at a depth of 20 mm for both tumours was also larger than that measured by^19^ (PVDR of 5.5 in a water phantom) with passive scattering and a ctc distance of 3.2 mm. The high PVDR, higher than in our previous small animal experiments^4,5^, and the low valley doses should enhance normal tissue sparing. Increasing the ctc distance from 4 mm to 6 mm approximately doubled the PVDR at the entrance for both tumours. The PVDR for the meningioma was 1.1 ± 0.2 for plan 3 and 1.6 ± 0.2 for plan 4 at a distance of 5 mm from the PTV (at a depth of 90 mm). The mean PVDR for the glioma, evaluated 5 mm from the PTV (at a depth of 90 mm), was 1.1 ± 0.1 for plan 3 and 1.4 ± 0.2 for plan 4.

**Table 4:** Examples of minibeam size (FWHM), PVDR and valley doses evaluated at different depths for treatment plans 3 and 4. The PVDR uncertainties are the standard deviation of the PVDR, as defined in section II.3.

Depth (mm)	ctc: 4 mm			ctc: 6 mm		
	Beam size (mm)	PVDR	Valley dose (%)	Beam size (mm)	PVDR	Valley dose (%)
**(a) Glioma (field 2)**						
1	0.7	19.2 ± 1.5	6.2	1.0	29.1 ± 1.9	6.2
10	0.7	17.5 ± 0.6	6.5	1.1	29.1 ± 0.9	6.5
20	0.9	12.8 ± 0.8	7.1	1.2	23.9 ± 1.3	6.9
40	1.2	7.1 ± 0.7	7.5	1.6	16.2 ± 1.4	7.1
60	1.7	3.6 ± 0.2	8.3	2.0	10.8 ± 0.9	12
80	2.1	1.2 ± 0.1	71	3.1	1.5 ± 0.1	61
90	2.2	1.1 ± 0.1	93	3.2	1.4 ± 0.2	84
	**ctc: 4 mm**			**ctc: 6 mm**		
**Depth (mm)**	**Beam size (mm)**	**PVDR**	**Valley dose (%)**	**Beam size (mm)**	**PVDR**	**Valley dose (%)**
**(b) Meningioma (field 1)**						
1	0.7	16.8 ± 2.3	5.2	0.8	44 ± 18	5.1
10	0.7	12.4 ± 1.5	5.8	0.8	35 ± 1	5.3
20	0.8	9.2 ± 0.4	6.1	0.8	22.6 ± 0.7	5.5
40	1.2	5.2 ± 0.2	7.0	1.5	13.6 ± 0.4	5.7
60	2.0	2.4 ± 0.1	9.1	2.6	7.0 ± 0.3	6.5
80	2.3	1.3 ± 0.1	13.5	3.2	2.9 ± 0.1	21
90	2.4	1.1 ± 0.2	73	3.2	1.6 ± 0.2	65

**Figure 3:**
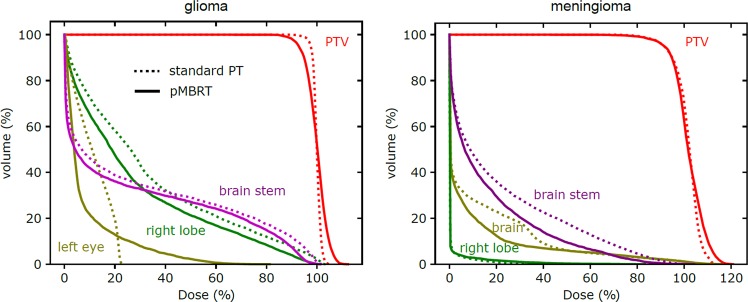
Comparison of dose-volume histograms for plan 2 (standard PT, dashed line) and plan 3 (pMBRT, solid line) for both glioma and meningioma. OAR close to the patient’s surface, such as the left eye or the brain, received a lower average dose with pMBRT than with standard PT.

The cumulative DVH for plan 2 (standard PT) and plan 3 (pMBRT) are displayed in Fig. 3 for certain relevant organs/structures. Although the PTV coverage was slightly better with standard PT than with pMBRT, the volumes (%) that receives more than 110% and less than 93% of the prescribed dose for glioma case were quite similar (see dosimetric values in Table 3a). Further optimisation of the standard plan and ctc values would have been necessary to improve these values for the meningioma. Moreover, standard and pMBRT plans resulted in similar DVHs for deep-seated organs-at-risk (OAR), such as the brainstem or right lobe. In contrast, the DVH for shallow organs, such as the left eye, extended to include higher doses in the case of pMBRT. For these sites, pMBRT results in an overall decrease of the average dose received (from 21.5% to 15.7% for the brain, and from 9.7% to 8.4% for the left eye).

**Figure 4:**
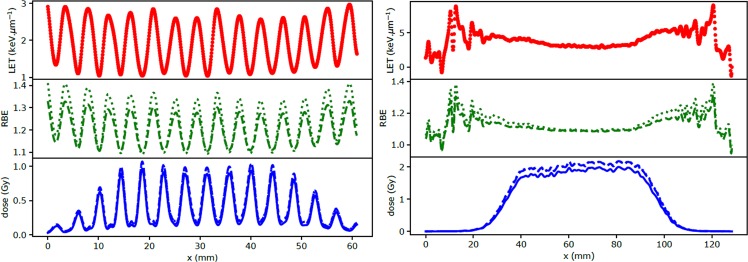
LET, RBE and dose distributions measured in healthy tissues at a depth of approximately 5 cm (left) and in the PTV (right) (meningioma, field 1). The RBE is calculated using the models described in reference^18^ (green dashed line) and^17^ (green dotted line) with a dose per fraction set at 2 Gy and an α/β parameter equal to 3 Gy (healthy brain tissues) and 10 Gy (PTV). The physical dose obtained directly from the simulation is displayed as a blue solid line and the RBE weighted dose is displayed in blue for both models (dashed and dotted lines).

LET and dose distributions measured in healthy tissues (at approximately mid-range) and in the PTV are displayed in Fig. 4 for the meningioma (field 1). Variations in the LET distribution from 1 to 3 keV.µm^−1^ were observed between the peak and valley regions in healthy tissues, due to the fact that only scattered and secondary particles contribute to the valley regions. The higher LET/RBE values observed outside of the PTV (Fig. 4, right) are due to scattered, lower energy secondary protons, which are the main contributors to the low-dose lateral penumbra^20,21^. The RBE distributions weighted by the dose distributions are displayed in blue for both models (dashed and dotted lines) in order to overcome this statistical effect.

Similarly, the RBE distribution varied between 1.05 and 1.4 in healthy tissues, significantly different from the spatially invariant RBE value of 1.1 commonly used in clinical practice. The LET in the PTV ranged between 3 and 5 keV.µm^−1^, which corresponds to an RBE ranging from 1.07 to 1.1 (for both RBE models).

## Discussion and conclusions

pMBRT is a novel method that appears to be promising to increase the therapeutic index for radioresistant tumours. The good results obtained in small animal experiments^5,6^, together with recent implementation of pMBRT in the ICPO PBS system^7^, were the basis for this evaluation of possible patient treatment plans.

To our knowledge, this is the first study on anonymized patients in realistic clinical settings. For this first investigation, we defined a homogeneous dose in the target as a constraint, corresponding to the conditions for which the best results in terms of long-term survival have been obtained^6^. Our evaluation shows that, by designing a suitable collimator, (quasi)homogeneous doses (PVDR < 1.2), with s-indices ranging from 5.5% (glioma) to 7.0% (meningioma), can be feasibly delivered to a deep-seated target volume by proton minibeam radiotherapy. It is also noteworthy that very good results in terms of tumour control have been obtained with pMBRT with a PVDR of 1.2 in the target compared to standard PT^6^. Consequently, plan 4, with larger ctc distances and a less uniform coverage of the tumour could also be a good alternative for treatment, as high PVDR are obtained at the entrance.

It should be stressed that good homogeneity can be achieved in large target volumes, while maintaining high spatial dose fractionation at shallow depths, which would simplify treatment and reduce potential errors. The PVDR in normal tissues (see Table 4) was situated in the same range or was higher than those used in small animal experiments, in which good normal tissue sparing was obtained^4,5,19^. The valley doses in the first 6 cm are less than 10% of the prescribed dose. Since more biological data are needed to establish the pMBRT parameters that optimise the therapeutic index, a direct comparison was considered to be the fairest approach at this stage. However, we do not expect any change in terms of the selection of field directions and numbers. The field arrangements are selected in that way in seamless proton therapy as to avoid complex tissue interfaces or critical OAR (the gradient at the distal end of proton dose distributions is rarely used to spare critical normal tissues due to uncertainties about its exact position in the patient), and we do not expect pMBRT would change these practices.

It should be stressed that there is still room for improvement, as the weights of the PBS spots were optimized for a seamless delivery. The PVDR in normal tissues close to the PTV could be increased by developing dedicated spot optimization algorithms taking into account the geometry of the collimator (ctc and slit width) shaping the specific Bragg peak of minibeams. Moreover, pMBRT resulted in a significant reduction of the average relative doses received by shallow OAR compared to conventional PT. Similar DVHs were obtained for deep-seated organs for both pMBRT and standard PT, but several OARs were more effectively spared by spatial dose fractionation in pMBRT. This study was designed to assess relative dose distributions in intracranial targets, neglecting the interdependencies between (hypo)fractionation and spatial dose distribution: due to the lack of clinical data for this type of experimental treatment, the quality of OAR sparing may indeed depend on the prescribed dose to the PTV (expressed as a percentage in this study) and the dose fractionation scheme adopted for treatment, resulting in different absolute valley doses, which can significantly affect the OAR response.

In conclusion, this proof of concept study shows that pMBRT may provide satisfactory treatment plans for brain tumour patients with only one or two proton minibeam arrays delivered by an existing set-up using a multislit collimator at a clinical centre. The dose distribution in the target complies with the standard criteria, while the spatial fractionation in normal tissues might significantly increase the therapeutic index.

Further investigations on more patients, including different tumour sites, will be performed to support the design of future clinical research.

## Data Availability

The datasets generated and/or analysed during this study are available from the corresponding author on reasonable request.
